# Hip MRI in flexion abduction external rotation for assessment of the ischiofemoral interval in patients with hip pain—a feasibility study

**DOI:** 10.1186/s13244-023-01524-4

**Published:** 2023-10-15

**Authors:** Alexander F. Heimann, Jonas Walther, Moritz Tannast, Joseph M. Schwab, Moritz Wagner, Alexander Brunner, Till D. Lerch, Simon D. Steppacher, Peter Vavron, Ehrenfried Schmaranzer, Florian Schmaranzer

**Affiliations:** 1 Department of Orthopaedic Surgery, HFR – Cantonal Hospital, University of Fribourg, Chemin des pensionnats 2 – 6, CH-1700 Fribourg, Switzerland; 2 Department of Orthopaedic Surgery, Inselspital Bern, University Hospital, University of Bern, Freiburgstrasse 18, Bern, CH-3010 Switzerland; 3 Department of Orthopaedic Surgery, District Hospital St. Johann in Tirol, Bahnhofstraße 14, 6380 St. Johann in Tirol, Austria; 4 Department of Diagnostic-, Interventional- and Pediatric Radiology, Inselspital Bern, University Hospital, University of Bern, Freiburgstrasse 18, Bern, CH-3010 Switzerland; 5 Department of Radiology, District Hospital St. Johann in Tirol, Bahnhofstraße 14, St. Johann in Tirol, 6380 Austria; 6 Department of Radiology, Balgrist University Hospital, Forchstrasse 340, Zurich, CH-8008, Switzerland

**Keywords:** Hip, Femoroacetabular impingement, Magnetic resonance imaging, Hip arthroscopy, Ischiofemoral impingement

## Abstract

**Objectives:**

To assess the feasibility of flexion-abduction-external rotation (FABER) magnetic resonance imaging (MRI) of the hip to visualize changes in the ischiofemoral interval and ability to provoke foveal excursion over the acetabular rim.

**Methods:**

IRB-approved retrospective single-center study. Patients underwent non-contrast 1.5-T hip MRI in the neutral and FABER position. Two readers measured the ischiofemoral interval at three levels: proximal/distal intertrochanteric distance and ischiofemoral space. Subgroup analysis was performed for hips with/without high femoral torsion, or quadratus femoris muscle edema (QFME), respectively. A receiver operating curve with calculation of the area under the curve (AUC) for the prediction of QFME was calculated. The presence of foveal excursion in both positions was assessed.

**Results:**

One hundred ten patients (121 hips, mean age 34 ± 11 years, 67 females) were evaluated. FABER-MRI led to narrowing (both *p* < .001) of the ischiofemoral interval which decreased more at the proximal (mean decrease by 26 ± 7 mm) than at the distal (6 ± 7 mm) intertrochanteric ridge. With high femoral torsion/ QFME, the ischiofemoral interval was significantly narrower at all three measurement locations compared to normal torsion/no QFME (*p* < .05). Accuracy for predicting QFME was high with an AUC of .89 (95% CI .82–.94) using a threshold of ≤ 7 mm for the proximal intertrochanteric distance.

With FABER-MRI foveal excursion was more frequent in hips with QFME (63% vs 25%; *p *= .021).

**Conclusion:**

Hip MRI in the FABER position is feasible, visualizes narrowing of the ischiofemoral interval, and can provoke foveal excursion.

**Critical relevance statement:**

FABER MRI may be helpful in diagnosing ischiofemoral impingement and detecting concomitant hip instability by overcoming shortcomings of static MR protocols that do not allow visualization of dynamic changes in the ischiofemoral interval and thus may improve surgical decision making.

**Key points:**

• FABER MRI enables visualization of narrowing of the ischiofemoral interval proximal to the lesser trochanter.

• Proximal intertrochanteric distance of ≤ 7 mm accurately predicts quadratus femoris muscle edema.

• Foveal excursion was more frequent in hips with quadratus femoris muscle edema.

**Graphical Abstract:**

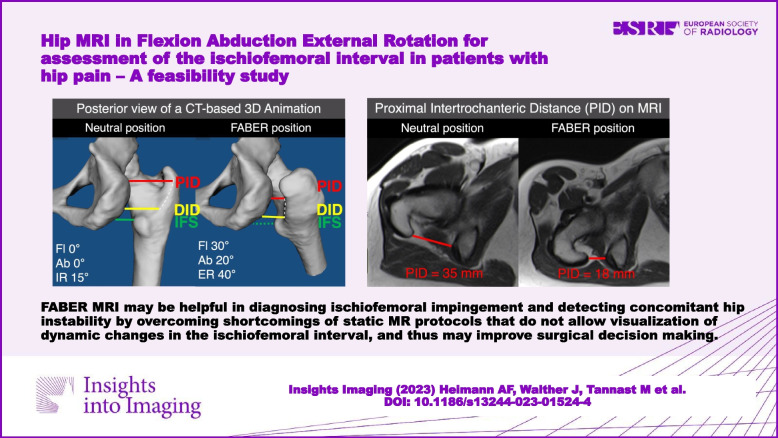

**Supplementary Information:**

The online version contains supplementary material available at 10.1186/s13244-023-01524-4.

## Introduction

Ischiofemoral impingement (IFI) is a cause of hip pain in young adults due to a mechanical conflict between the proximal femur and ischium, initially described between the lesser trochanter and ischial tuberosity [1]. It is more common in women and is bilateral in one third of cases [2, 3]. Patients usually present with hip and buttock pain and a positive posterior impingement test (pain with combined extension-adduction-external rotation). In addition, a positive FABER test (pain with combined flexion-abduction-external rotation) also suggests IFI [4]. These provocative tests lead to narrowing of the ischiofemoral interval and can provoke pain in the buttocks and groin. This is supposedly related to the abutment of the quadratus femoris muscle (QFM) and increased stress on the anterior chondro-labral junction by leverage of the femoral head and maltracking of the fovea capitis [5]. Furthermore, many patients present with additional hip deformities such as femoroacetabular impingement (FAI) or developmental hip dysplasia and chondro-labral lesions making clinical diagnosis of IFI difficult [5, 6].

The pathomechanics of IFI are poorly understood. Research into potential pathomechanisms have identified abductor insufficiency [7], increased femoral antetorsion, and valgus deformity [8–10] as contributing factors. This has, in turn, led to the proposal of different treatment strategies.

Magnetic resonance imaging (MRI) with fluid-sensitive sequences is vital in diagnosing IFI as it shows the narrowing of the ischiofemoral space (IFS) and QFM edema (QFME) [3, 11]. However, current static MR imaging protocols do not directly visualize changes in the ischiofemoral interval. To overcome this limitation, we introduce an MRI protocol in which the hip is positioned in flexion, abduction, and external rotation (FABER) similar to the clinical test.

The aim of this study was to assess (1) the feasibility of performing FABER-MRI of the hip to visualize changes in the ischiofemoral interval, (2) changes in the ischiofemoral interval in normal and FABER positions depending on high/normal femoral torsion and/ or the presence of QFME, and (3) the relationship between the fovea capitis and the acetabular rim during FABER-MRI.

## Materials and methods

### Study design and participant inclusion

Institutional review board (IRB) approved a single-center, retrospective observational study conducted at a primary referral center for joint preserving hip surgery in Austria. The study was performed with a written informed consent waiver.

**Fig. 1:**
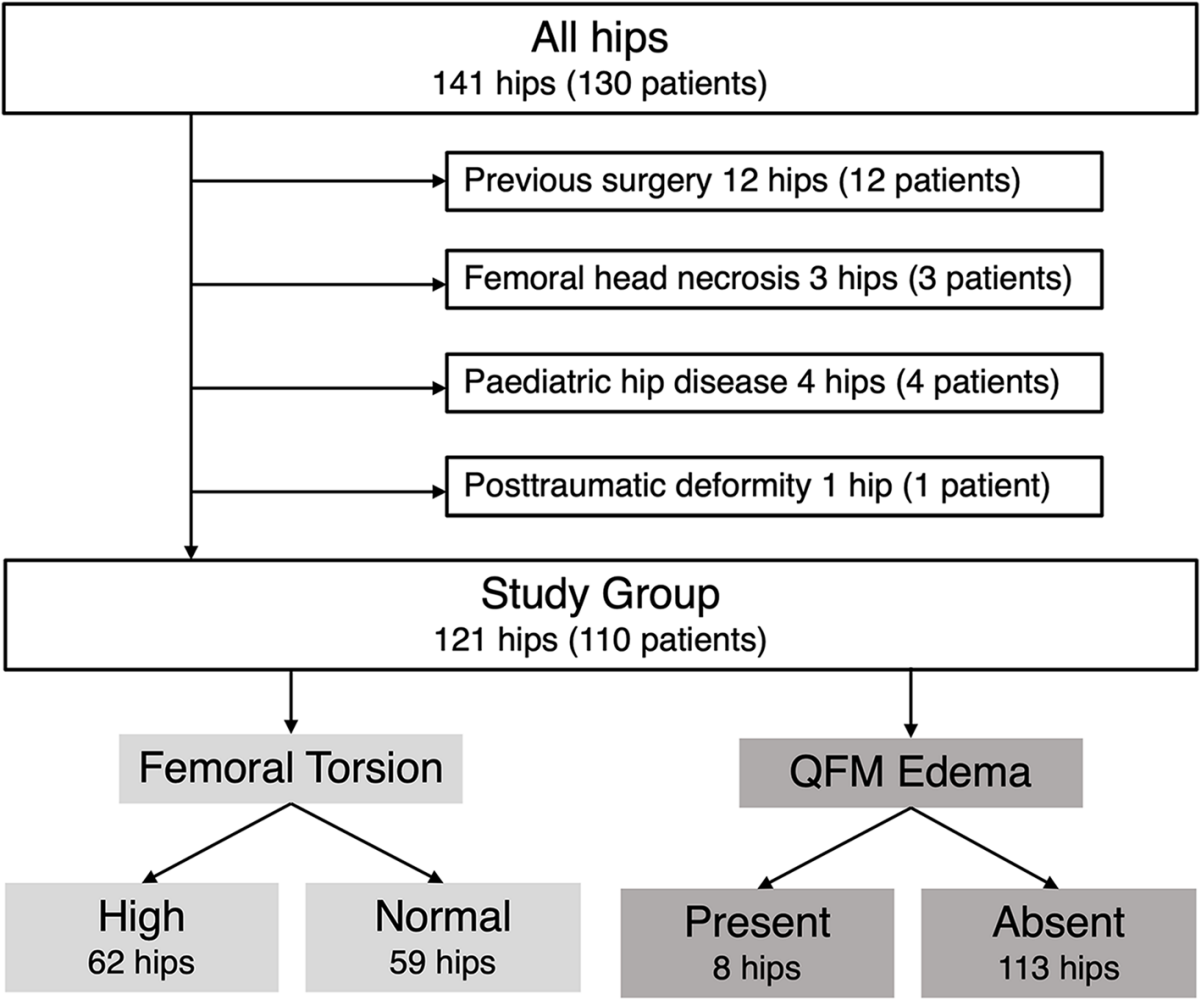
Study flowchart. QFM, quadratus femoris muscle

We included a consecutive series of patients who consulted our outpatient orthopedic clinic for hip and/ or buttock pain between October 2019 and September 2020. All patients underwent diagnostic imaging of the symptomatic hip. Exclusion criteria were previous hip surgery, femoral head necrosis, pediatric hip disease, or posttraumatic deformity. The overall study cohort was 121 hips (110 patients) which was divided into increased (> 30°) and normal/low (≤ 30°) femoral torsion and by presence or absence of QFME (Fig. 1).

### Radiographic imaging and hip MR arthrography

All patients underwent anteroposterior pelvis radiographs and 45° modified Dunn views of the affected hip according to a standardized acquisition technique [12].

All patients underwent 1.5-T MR arthrography (Magnetom Aera, Siemens Healthineers) with intra-articular contrast under fluoroscopic guidance. The protocol included the acquisition of axial short-tau inversion recovery (STIR) and 3D T1-weighted volumetric interpolated breath-hold examination DIXON sequences of the pelvis and distal femoral condyles. Multiplanar proton-density weighted turbo spin echo images in coronal, axial-oblique, sagittal, and radial orientation, using a standardized limb traction technique [13, 14], were acquired to detect chondro-labral lesions. Sequence protocol details are shown in Supplementary Table 1.

### FABER-MRI of the hip

**Fig. 2:**
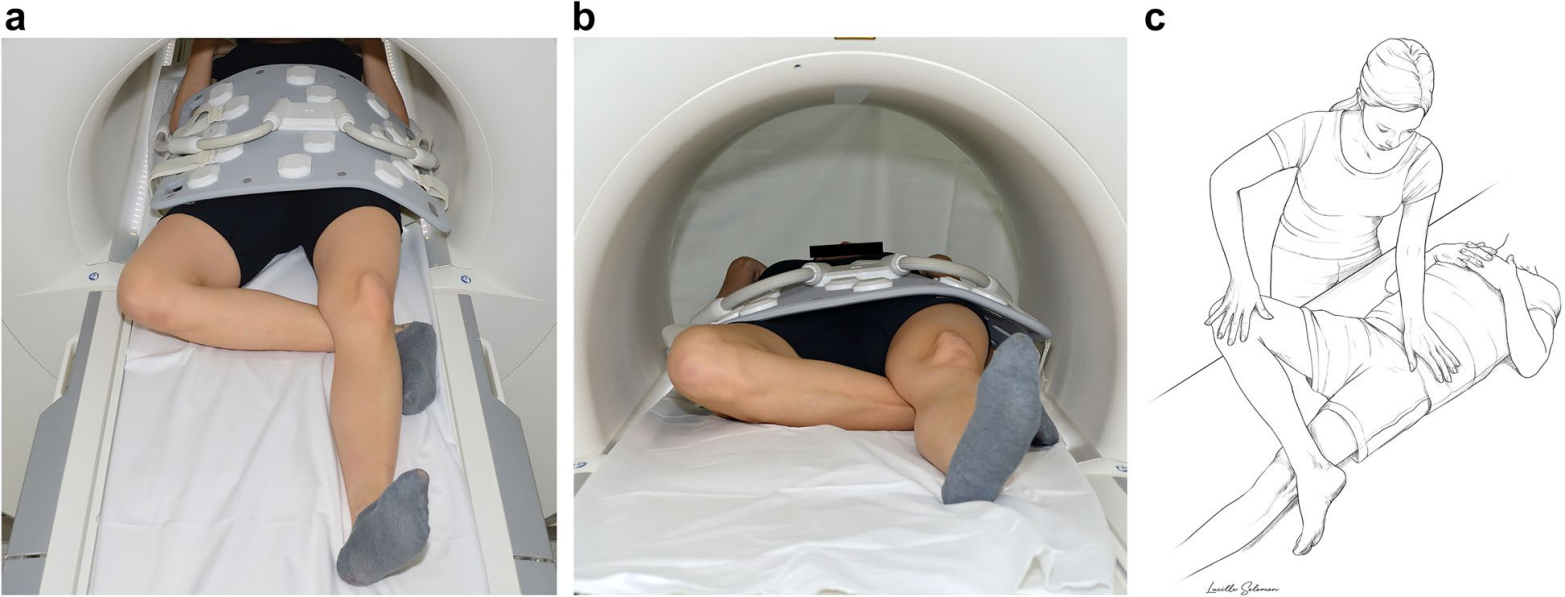
Illustration of patient positioning in the FABER stress position. **a** Top view. **b** Front view. This positioning mimics the (**c**) clinical FABER test (flexion-abduction-external rotation test)

Non-contrast MRI of the hip in the neutral and FABER positions was scheduled as a subsequent appointment to the initial MR arthrography. The mean time interval between the two examinations was 2 ± 2 days (range 1 to 13 days). MRI was performed at the same 1.5-T scanner (Magnetom Aera, Siemens Healthineers), with a 70-cm gantry using a large flexible body coil. Imaging in neutral position was performed first with the feet fixed in 15° of internal rotation. This was followed by technician repositioning of the affected leg in slight flexion, abduction, and external rotation (FABER position). The ankle of the affected leg was positioned under the contralateral knee joint to provide stability (Fig. 2). Axial T2-weighted half-Fourier-acquired single-shot turbo spin echo sequence (HASTE) of the pelvis (acquisition time, 39 s) and an axial-oblique T2-weighted 2D true fast imaging with steady-state free precession (True FISP) sequence (acquisition time, 51 s) of the affected hip were acquired (Supplementary Table 2). Both sequences were acquired in the neutral and FABER positions with an overall imaging time, including positioning, of 5–7 minutes.

### Image analysis

Analysis was performed by a radiologist with 12 years of hip imaging experience (E.S.). This included measurements recommended by the Lisbon agreement on FAI assessment including acetabular coverage (lateral center edge angle), signs of acetabular retroversion (cross-over, posterior wall and ischial spine sign), and measurement of femoral neck-shaft angle [15] (Table 1).

**Table 1:** Demographic and radiographic parameters

**Parameter**	**Overall** (*N* = 121)	**Femoral torsion**^a^			**QFM edema**		
		High (*N *= 62)	Normal (*N *= 59)	*p*	Present (*N *= 8)	Absent (*N *= 113)	*p*
Sex, female	67 (55)	44 (71)	23 (39)	< .001*	8 (100)	59 (52)	.009*
Age, years	34 ± 11	34 ± 12	34 ± 11	.697	40 ± 10	34 ± 12	.124
Side, right	61 (50)	31 (50)	30 (51)	.926	3 (38)	58 (51)	.452
LCE-Angle, °	29 ± 8	31 ± 9	28 ± 8	.006*	31 ± 7	29 ± 9	.480
Cross-over sign, +	30 (25)	15 (24)	15 (25)	.876	2 (25)	28 (25)	.989
Posterior wall sign, +	51 (42)	21 (34)	30 (51)	.060	4 (50)	47 (42)	.643
Ischial spine sign, +	28 (23)	15 (24)	13 (22)	.779	2 (25)	26 (23)	.898
Acetabular version, °	18 ± 6	18 ± 6	17 ± 6	.193	17 ± 5	18 ± 6	.795
Ischial angle, °	128 ± 11	129 ± 5	126 ± 15	.142	130 ± 3	128 ± 12	.277
Neck shaft angle, °	129 ± 5	131 ± 5	129 ± 5	.060	132 ± 5	130 ± 5	.318
Femoral torsion, °	30 ± 10	39 ± 6	23 ± 6	< .001*	42 ± 10	30 ± 9	< .001*
Cam, alpha angle >60°	66 (55)	31 (50)	35 (59)	.012*	2 (25)	66 (11)	.028*
Subsequent surgery	32 (26)	17 (27)	15 (25)	.804	5 (63)	27 (24)	.017*

^a^High and normal femoral torsion are defined as > 30°/≤ 30° according to Murphy [16, 17]

Numerical data are mean ± SD. Categorical data are *N* (%). *QFM(E)*, quadratus femoris muscle (edema); *SD*, standard deviation. *p < .05

Angular measurements on MRI were performed on axial 3D T1-weighted volume interpolated breath-hold-examination (VIBE) DIXON sequences, which were part of the MR arthrography protocol. Femoral torsion was measured using the method described by Murphy et al. [16, 17]. The study cohort was then divided into patients with increased (> 30°) and normal/ low (≤ 30°) femoral torsion. Acetabular version was measured at the midlevel of the femoral head [18]. The ischial angle was measured to assess inter-ischial distance [1]. The presence of QFME was defined as increased signal intensity in the QFM on axial STIR images and the study group was divided into patients with and without QFME.

### Assessment of FABER-MRI

**Fig. 3:**
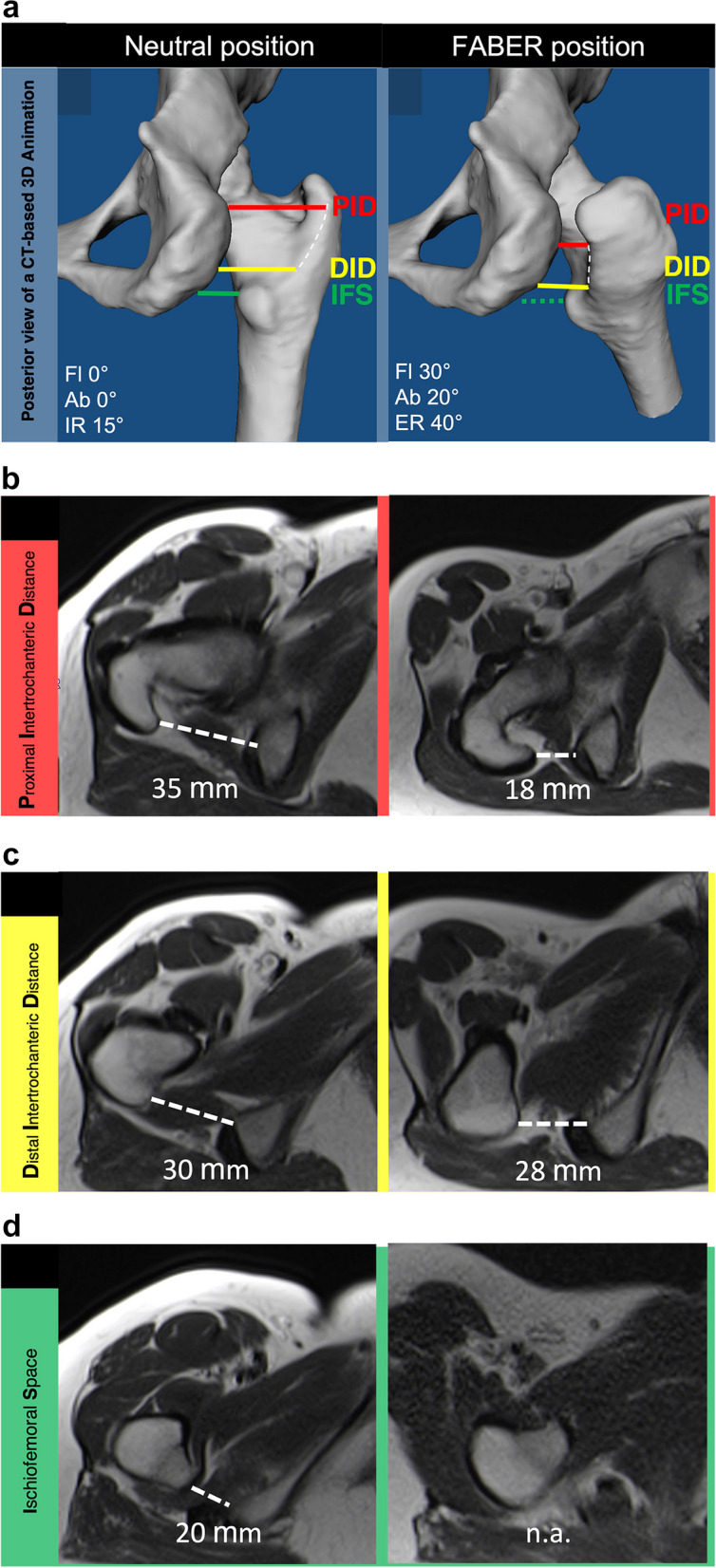
**a** Posterior view of a CT-based 3D animation of a right hip joint in neutral (left) and FABER (right) positions with the corresponding MRI images measuring the (**b**) proximal intertrochanteric distance (PID, red line) and (**c**) distal intertrochanteric distance (DID, yellow line), as well as the (**d**) ischiofemoral space (IFS, green line). The white dashed line in the 3D animation marks the intertrochanteric ridge. Fl, flexion. Ab, abduction. IR, internal rotation. ER, external rotation. n.a.,= not applicable

The ischiofemoral interval was analyzed on axial T2-weighted HASTE images in both the neutral and FABER positions at three different levels (Fig. 3):

- *Proximal intertrochanteric distance (PID):* The shortest distance between the lateral cortex of the ischial tuberosity and the most proximal point of the intertrochanteric ridge.
- *Distal intertrochanteric distance (DID):* The shortest distance between the lateral cortex of the ischial tuberosity and the most distal point of the intertrochanteric ridge.
- *Ischiofemoral space (IFS):* The shortest distance between the lateral cortex of the ischial tuberosity and medial cortex of the lesser trochanter [1].

The neutral and FABER position measurements were compared for the overall cohort, followed by subgroup analysis in hips with/without increased femoral torsion and in hips with/ without QFME.

**Fig. 4:**
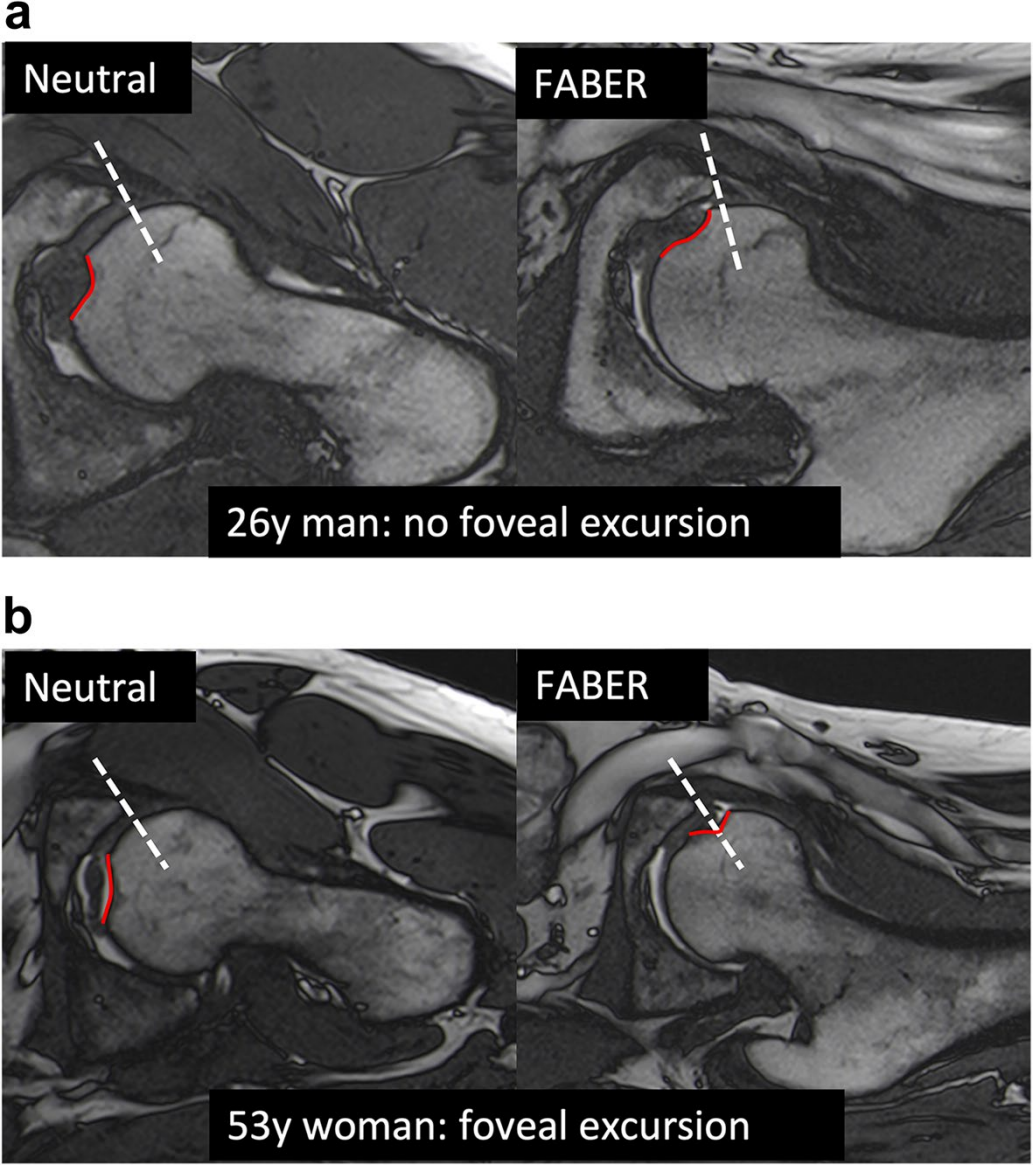
Assessment of foveal excursion on axial-oblique T2-weighted True FISP images. **a** No foveal excursion: In both neutral (left) and FABER (right) positions, the fovea capitis femoris (solid red line) is not crossed by a line perpendicular to the anterior acetabular rim (dashed white line). **b** Foveal excursion: The fovea capitis femoris (solid red line) is crossed by the line perpendicular to the anterior acetabular rim (dashed white line) in the FABER position

Foveal excursion extending over the acetabular rim was assessed as follows: intersection of the fovea capitis femoris with a line perpendicular to the anterior acetabular rim on the axial-oblique T2-weighted True FISP images (Fig. 4). This was determined in both the neutral and FABER positions. In addition, a subgroup analysis was performed in hips with/without high femoral torsion and in hips with/ without QFME.

A random sample of 48 hips was assessed by a second radiologist with 7 years of experience to evaluate both ischiofemoral interval and foveal excursion (F.S.). This number was based on a prior power analysis which yielded 46 hips to determine interobserver reliability with an intraclass correlation coefficient greater than 0.80 using an alpha error of 0.05 and a power of 80%.

### Statistical analysis

MedCalc® (MedCalc Statistical Software, version 20.106, MedCalc Software Ltd, Ostend, Belgium) was used for statistical analysis. The Kolmogorov-Smirnov test was performed for distribution testing of numerical data. Subgroup analysis of normally/not normally distributed data was performed using an unpaired Student’s *t*-test/Wilcoxon test. Changes in the ischiofemoral interval were compared with paired Student’s *t*-test. Binary data was tested using a chi-square test. Simple linear regression analysis and Pearson’s correlation coefficient r_p_ were used to correlate the relative change of the ischiofemoral interval and femoral torsion. To determine the diagnostic threshold of the measurements of the ischiofemoral interval for predicting QFME in the neutral and FABER positions, a receiver operating curve (ROC) was constructed and the area under the curve (AUC) was calculated. Interobserver agreement of the ischiofemoral interval and the presence of femoral excursion was calculated using interobserver correlation coefficient (ICC) and Cohen’s kappa (κ), respectively. Interpretation of interobserver agreement was performed as follows: ICC > 0.9 almost perfect, > 0.80 substantial, > 0.60 fair, and < 0.40 poor agreement [19]. With *ĸ* values of 0.81–1.00, rated consistent to almost perfect, 0.61–0.80 substantial, 0.41–0.60 moderate, 0.21–0.40 fair, and 0.01–0.20 none to slight agreement [20].

## Results

**Fig. 5:**
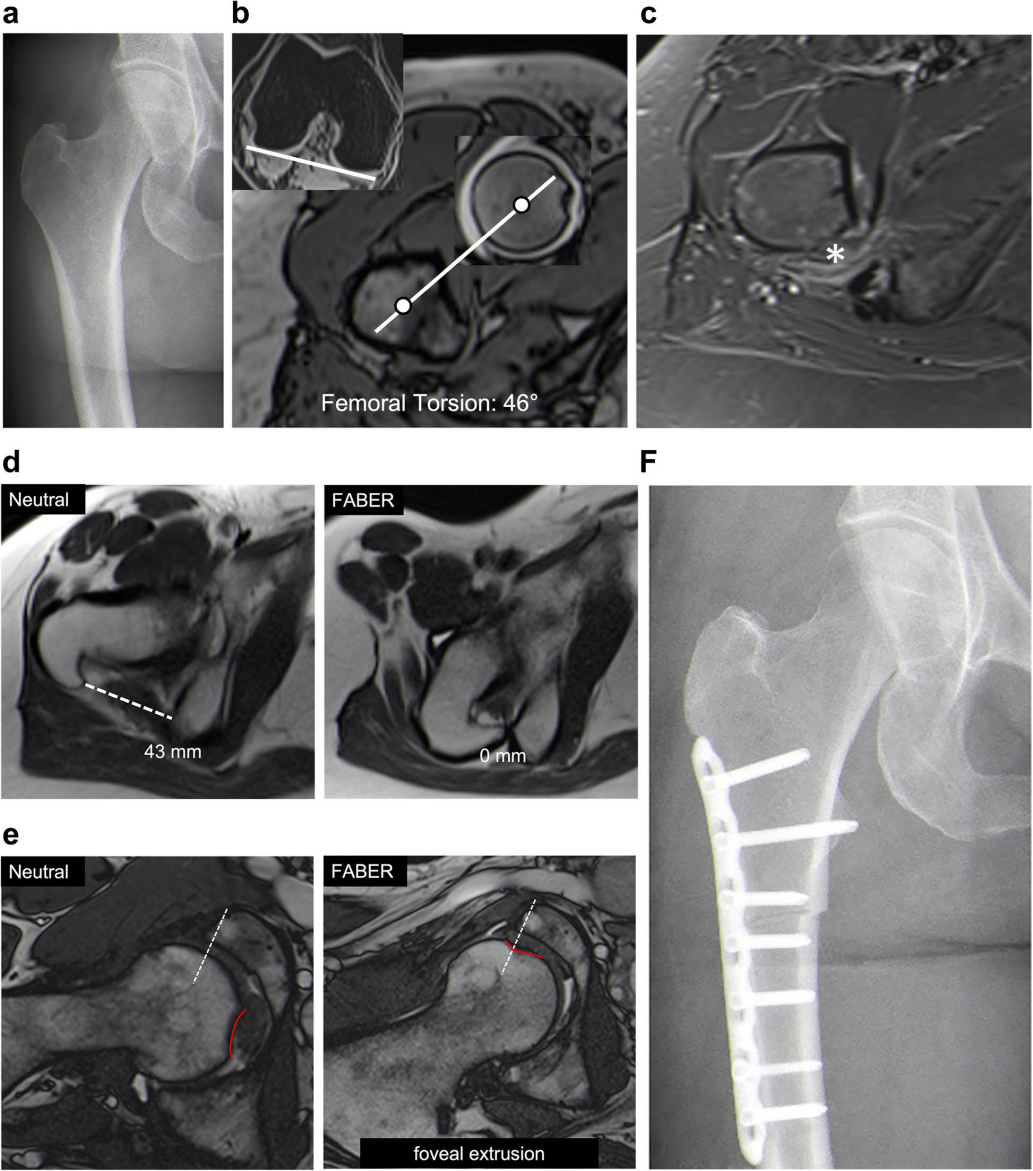
**a** Anteroposterior radiograph of the right hip of a patient with (**b**) increased femoral torsion of 46° according to Murphy and a (**c**) quadratus femoris muscle edema on axial STIR images. FABER-MRI revealed a reduction in the (**d**) proximal intertrochanteric distance from the neutral to the FABER position, in which (**e**) furthermore, a foveal excursion was evident. **f** Postoperative radiograph after subtrochanteric derotation osteotomy

From a total of 121 hips (110 patients) available for analysis, 55% were females. The mean age was 34 ± 11 years (Table 1). Thirty-two hips (26%) underwent subsequent surgery. Of these, 27 (84%) underwent hip arthroscopy. Four hips (12%) underwent either subsequent (2 patients) or additional (2 patients) subtrochanteric derotation osteotomy for the treatment of ischiofemoral impingement (Fig 5). One patient (3%) underwent primary total hip arthroplasty. All patients were able to complete the FABER-MRI protocol. Sixty-two hips (51%) had femoral torsion of > 30° and eight hips (7%) had QFME. Comparison between hip deformities most importantly revealed higher femoral torsion in patients with QFME (42 ± 10° vs 30 ± 9°, *p* < .001, Table 1).

### Changes in the ischiofemoral interval with FABER-MRI

Overall, the ischiofemoral interval decreased significantly between neutral and FABER positions at both the proximal (40 ± 8 mm versus 14 ± 9 mm, *p* < .001) and distal intertrochanteric measurement sites (28 ± 8 mm versus 21 ± 9 mm, *p* < .001) (Fig. 3a–c, Table 2). PID decreased significantly more (26 ± 7 mm vs 6 ± 7 mm,* p* < .001) and more frequently than DID (100% vs 78%, *p* < .001).

**Table 2:** Measurement of the ischiofemoral interval depending on femoral torsion and the presence of a quadratus femoris muscle edema on MRI

		**Femoral torsion**^a^			**QFM edema**		
	**Overall** (*N*= 121)	High (*N *= 62)	Normal (*N *= 59)	*p*	Present (*N *= 8)	Absent (*N *= 113)	*p*
**Proximal intertrochanteric distance** (mean in mm ± SD)							
Neutral position	40 ± 8	37 ± 8	43 ± 8	<.001*	30 ± 10	41 ± 8	.005*
FABER position	14 ± 9	11 ± 7	17 ± 9	.009*	4 ± 2	15 ± 9	< .001*
Mean difference	26 ± 7	26 ± 7	26 ± 8	.936	26 ± 9	26 ± 7	.831
**Distal intertrochanteric distance** (mean in mm ± SD)							
Neutral Position	28 ± 8	25 ± 7	31 ± 7	<.001*	20 ± 6	28 ± 7	.003*
FABER position	21 ± 9	19 ± 8	24 ± 10	.021*	13 ± 3	22 ± 9	.004*
Mean Difference	6 ± 7	6 ± 7	7 ± 8	.938	7 ± 7	6 ± 7	.871
**Ischiofemoral space** (mean in mm ± SD)							
Neutral position	27 ± 9	23 ± 8	31 ± 10	<.001*	17 ± 5	28 ± 9	< .001*
FABER position	n.a.	n.a.	n.a.	n.a.	n.a.	n.a.	n.a.

^a^High and normal femoral torsion are defined as > 30°/≤ 30° according to Murphy [16, 17]

Numerical data are mean ± SD. Categorical data are *N* (%). *QFM(E)*, quadratus femoris muscle (edema); *FABER*, flexion-abduction-external rotation; *n.a.*, not applicable. *p < .05

IFS was 27 ± 9 mm in the neutral position and not measurable in the FABER position. This was due to the lesser trochanter being more anterior and distal relative to the ischial tuberosity (Fig. 3d).

### Subgroup analysis of changes in the ischiofemoral interval with FABER-MRI

Changes in the ischiofemoral interval as a function of femoral torsion and the presence of QFME are depicted in Table 2.

In patients with increased femoral torsion, PID was significantly narrower in the neutral (37 ± 8 mm versus 43 ± 8 mm, *p* < .001) and FABER position (11 ± 7 mm versus 17 ± 9 mm, *p* = .009) compared to patients without increased torsion. DID was also significantly narrower in patients with high femoral torsion compared to no normal femoral torsion in both the neutral (25 ± 7 mm versus 31 ± 7 mm, *p* < .001) and FABER positions (19 ± 8 mm versus 24 ± 10 mm, *p* = .021). There was a positive correlation between increasing femoral torsion and narrowing of the ischiofemoral interval at both the proximal (*r*_p_ = .41, *p* < .001) and distal (*r*_p_ = .38, *p* < .001) intertrochanteric measurement location.

In hips with QFME, PID was significantly narrower in both the neutral (30 ± 10 mm versus 41 ± 8 mm, *p* = .005) and FABER positions (4 ± 2 mm versus 15 ± 9 mm, *p* < .001) compared to hips without edema. DID was also significantly narrower in hips with QFME in both the neutral (20 ± 6 mm versus 28 ± 7 mm, *p* = .005) and FABER positions (13 ± 3 mm versus 22 ± 9 mm, *p* = .004).

IFS was significantly narrower (all *p* < .001) in patients with high femoral torsion/QFME (23 ± 8 mm/17 ± 5 mm) compared to patients with normal femoral torsion/ without QFME (31 ± 10 mm/28 ± 9 mm).

**Fig. 6:**
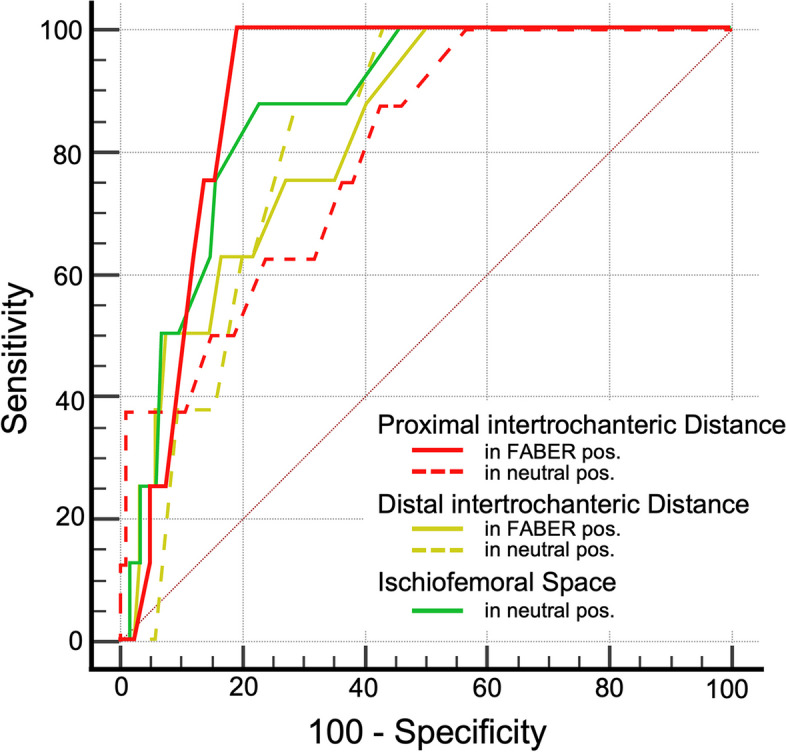
Receiver operating curves of the ischiofemoral interval at the different levels in both the neutral and FABER positions for prediction of a quadratus femoris muscle edema

The ROC curves were plotted (Fig. 6) and values with corresponding AUC are shown in Table 3. Accuracy was high for predicting QFME based on ischiofemoral interval measurements and ranged from .80 (95% CI .72–.87) for PID in the neutral position (threshold of ≤ 38 mm) to .89 (95% CI .82–.94) for PID in the FABER position (threshold of ≤ 7 mm). Applying a threshold of ≤ 20 mm for the IFS in a neutral position, the accuracy of predicting QFME was .87 (95% CI .73–.92).

**Table 3:** Diagnostic thresholds and associated parameters for predicting quadratus femoris muscle edema

	**Threshold**	**Sensitivity** (%)	**Specificity** (%)	**AUC**	**95% CI**
**Proximal intertrochanteric distance**					
In FABER position	≤ 7 mm	100	81	.89	.82–.94
In neutral position	≤ 38 mm	88	58	.80	.72–.87
**Distance intertrochanteric distance**					
In FABER position	≤ 15 mm	88	71	.81	.73–.88
In neutral position	≤ 28 mm	100	49	.81	.73–.88
**Ischiofemoral space**					
In neutral position	≤ 20 mm	88	77	.87	.73–.92

*AUC* Area und the curve, *CI* Confidence interval, *FABER* Flexion-abduction-external rotation

### Prevalence of foveal excursion in neutral position and FABER-MRI

No case of foveal excursion extending over the acetabular rim was observed in the neutral hip position. This increased to 27% on FABER-MRI (*p* < .001) (Table 4). Foveal excursion was significantly more frequent in hips with QFME on FABER-MRI than in patients without edema (63% vs 25%, *p* = .021).

**Table 4:** Prevalence of foveal extrusion depending on femoral torsion and the presence of a quadratus femoris muscle edema in the MRI

		**Femoral torsion**^a^			**QFM Edema**		
	**Overall** (*N *= 121)	High (*N *= 62)	Normal (*N *= 59)	*p*	Present (*N *= 8)	Absent (*N *= 113)	*p*
Neutral position	0 (0)	0 (0)	0 (0)	> .99	0 (0)	0 (0)	> .99
FABER position	33 (27)	17 (27)	16 (27)	.657	5 (63)	28 (25)	.021*

High and normal femoral torsion are defined as > 30°/≤ 30° according to Murphy [16, 17]

Data are *N* (%). *QFM(E)*, Quadratus femoris muscle (edema), *FABER* Flexion-abduction-external rotation. *p < .05

### Interobserver agreement

Interobserver agreement for measurement of the ischiofemoral interval was substantial to almost perfect ranging from an ICC of .86 for DID in neutral position to ICC of .96 for PID in the FABER position (Table 5). Interobserver agreement on the presence of foveal excursion over the acetabular rim was almost perfect with a *κ* value of .90 (95% CI .76–1.0, Table 5).

**Table 5:** Interrater agreement

	**ICC**	**95% CI**
**Neutral Position**		
Proximal Intertrochanteric Distance	.93	.87–.96
Distal Intertrochanteric Distance	.86	.74–.92
**FABER Position**		
Proximal Intertrochanteric Distance	.96	.92–.98
Distal Intertrochanteric Distance	.92	.85–.96
Foveal excursion in FABER position	**Cohen’s κ**	**95% CI**
	.90	.76–1.0

*ICC* Interobserver correlation coefficient, *FABER* Flexion-abduction-external rotation

## Discussion

This pilot study demonstrates the feasibility of FABER-MRI of the hip. This examination with fast image acquisition (5 to 7-min acquisition time) is adapted from the clinically used FABER test. Narrowing between the lesser trochanter and the ischial tuberosity was not observed in any of the FABER-MRIs we performed. By contrast, FABER-MRI allowed for visualization of narrowing in the ischiofemoral interval more proximally. At the level of the intertrochanteric ridge, narrowing was more pronounced proximally (mean decrease of 26 mm) than distally (range 6 to 7 mm). The narrowest ischiofemoral interval was observed proximally at the PID (4 ± 2 mm in FABER- vs 30 ± 10 mm in neutral position) in patients with QFME. Accordingly, a PID ≤ 7 mm on FABER-MRI accurately predicted QFME with an AUC .89 (95% CI .82–.94). These results indicate that posterior extraarticular impingement in IFI may occur when either the posterior portion of the greater trochanter or the intertrochanteric ridge impinges on the ischial tuberosity. In addition, one in four patients (25%) demonstrated foveal excursion over the acetabular rim on FABER-MRI. This prevalence increased to 63% in hips with QFME. This supports the hypothesis that in IFI posterior extraarticular impingement may provoke hip instability by causing anterior femoral head levering with maltracking of the fovea capitis [5, 21].

IFS dimensions reported from the literature range from 13 ± 5 mm to 17 ± 6 mm in patients with QFME [1, 3, 22] with higher values reported in controls ranging from 23 ± 8 mm to 31 ± 9 mm [1, 3, 23, 24]. This is comparable to our study in which IFS was lower in hips with QFME (17 ± 5 mm vs 28 ± 9 mm).

Previous attempts to improve the understanding of the pathomechanics in IFI using MRI have demonstrated dynamic, position-dependent changes [25, 26]. Vincentini et al. investigated the effect of external rotation on IFS dimension with kinematic MRI. When going from internal rotation to external rotation both the control group (34 ± 4 mm to 20 ± 3 mm) and the IFI group (28 ± 6 mm to 11 ± 5 mm) demonstrated IFS narrowing. The authors concluded that kinematic MRI detected dynamic differences of IFS with respect to the final position of the lesser trochanter [25]. Li et al. investigated the dynamic effect of the long stride walking test on IFS in a prospective MRI study of 37 patients with clinically diagnosed IFI and 39 healthy controls. They combined various uniaxial and biaxial movements in the supine and prone positions. They reported narrowing of the IFS in the supine position when moving from 30° internal rotation (25 ± 7 mm) to combined adduction and external rotation (11 ± 3 mm). In addition, they also reported narrowing of the IFS in the prone position during 30° extension (9 ± 3 mm) [26].

In contrast to the aforementioned studies, we aimed to adapt one of the two established clinical tests used for the diagnosis of IFI for MRI. The FABER test was chosen since the posterior impingement test would require prone patient positioning which would be time-consuming and difficult to standardize. However, it is important to note that this test is not specific for IFI and, like most clinical tests, has only moderate diagnostic efficacy. In the clinical setting, the FABER test is completed by applying progressive force to the knee to determine whether the test is positive or negative. FABER-MRI of the hip was feasible, and in our study, no patient requested termination of the examination. Interestingly, in the FABER position, the lesser trochanter moved more anteriorly and “out of plane” which prevented us from measuring the IFS. By contrast, we observed narrowing between the intertrochanteric ridge and the ischial tuberosity, especially at the proximal portion of the ridge, leading to an overall mean narrowing of 26 ± 7 mm (40 ± 8 mm vs 14 ± 9 mm) with FABER-MRI. In addition, we could identify a correlation between increasing femoral torsion and narrowing of the PID (*r*_p_ = .41) and DID (*r*_p_ = .38). Our results confirm the findings of a previous study using virtual, dynamic CT-based simulation of the FABER test using collision detection software. In that study, the intertrochanteric ridge and greater trochanter were identified as impingement location in 67% and 62%, respectively when simulating the FABER test in 40° of external hip rotation [27].

Furthermore, a PID of ≤ 7 mm in the FABER position predicted QFME with an accuracy similar to using ≤ 20 mm as threshold for the IFS. Thus, crush QFME could also result from impingement between the greater trochanter and/or the intertrochanteric ridge with the ischial tuberosity. This seems intuitive considering the fact that the footprint of the quadratus femoris muscle is located at the posteromedial aspect of the proximal femur at the intertrochanteric ridge [28].

Since the optimal surgical treatment of young patients with IFI is controversial [29] and ranges from resection of the lesser trochanter [30, 31] to femoral derotation osteotomy [5, 8], we believe that MRI visualization of the impingement location could be helpful for surgical decision making.

This study has limitations. First, we did not integrate the FABER-MRI into our institutional routine protocol. This was related to the fact that direct MR arthrography of the hip is routinely performed in our institution and there were concerns that intra-articular injection and subsequent FABER positioning may be too uncomfortable. However, we did not test this hypothesis nor did we implement the FABER position into a standard non-contrast MRI protocol of the hip. Since all study participants were able to complete the FABER-MRI examination it could be performed before the arthrography or be included into a non-contrast hip MRI examination in the future and thus be integrated more easily into clinical routine. Second, FABER-MRI was performed on a scanner with a 70-cm gantry. While image acquisition was feasible and tolerated throughout our study this may be more difficult when either using a scanner with a 60-cm gantry, or in a more obese population. Third, there were only 8 hips (7%) with QFME. Although this reflects the rarity of ischiofemoral impingement in the FAI population as a whole, these limitations do not allow us to draw conclusions about the actual clinical utility of FABER MRI. This emphasizes the need for future, prospective studies of patients undergoing subsequent surgery and appropriate follow-up to evaluate the added benefit of FABER-MRI in patients with suspected IFI. Fourth, unlike the clinical test in which progressive force is applied to the ipsilateral knee we decided to perform the MRI in a static FABER position. This position was considered easier to implement and to standardize than a dynamic motion protocol. However, dynamic assessment of hip motion using sequences with high temporal resolution would yield the potential advantage of a “real time” visualization of the impingement conflict at the maximum individual passive range of motion [28]. In conclusion, FABER-MRI of the hip is feasible, allows assessment of changes in the ischiofemoral interval proximal to the lesser trochanter, and detects foveal excursion. It enables the detection of “intertrochanteric type” IFI and possibly hip instability. We feel FABER-MRI has great potential to improve our understanding and surgical decision-making in IFI.

### Supplementary Information

**Additional file 1: Supplementary Table 1.** Imaging protocol for standard MR arthrography of the hip. **Supplementary Table 2.** Imaging protocol for MRI in neutral and FABER position.

## Data Availability

The datasets used and/or analyzed during the current study are available from the corresponding author upon reasonable request.

## Abbreviations

AUC: Area under the curve
DID: Distal intertrochanteric distance
FABER: Flexion-abduction-external rotation
FAI: Femoroacetabular impingement
FISP: Fast imaging with steady-state free precession
HASTE: Half-Fourier-acquired single-shot turbo spin echo sequence
ICC: Interobserver correlation coefficient
IFI: Ischiofemoral impingement
IFS: Ischiofemoral space
IRB: Institutional review board
MRI: Magnetic resonance imaging
PID: Proximal intertrochanteric distance
QFM(E): Quadratus femoris muscle (edema)
QFS: Quadratus femoris space
ROC: Receiver operating curve
STIR: Short-tau inversion recovery
VIBE: Volume interpolated breath-hold-examination
