# Supplementary material for: Hip MRI in flexion abduction external rotation for assessment of the ischiofemoral interval in patients with hip pain—a feasibility study
Source: Insights Imaging. 2023 Oct 15;14:172. doi: 10.1186/s13244-023-01524-4 (PMC10577115; doi:10.1186/s13244-023-01524-4)
Supplement: Supplementary file 1 — Additional file 1: Supplementary Table 1. Imaging protocol for standard MR arthrography of the hip. Supplementary Table 2. Imaging protocol for MRI in neutral and FABER position. [file 13244_2023_1524_MOESM1_ESM.docx]

| **Supplementary Table 1.** Imaging protocol for standard MR arthrography of the hip | | | | | | | | | |
| --- | --- | --- | --- | --- | --- | --- | --- | --- | --- |
| Sequence | Repetition  Time  (ms) | Echo  Time  (ms) | Inversion Time  (ms) | Matrix | FOV (mm) | Flip  Angle | Slice  Thickness (mm) | Bandwidth  (Hz/Px) | Image orientation |
| PD-w turbo^1^ spin echo | 2460 | 13 | - | 512 x 512 | 180 | 150 | 3 | 130 | coronal/ sagittal/ axial-oblique/ radial |
| STIR | 4360 | 42 | 150 | 384 x 384 | 360 | 150 | 5 | 161 | axial pelvis |
| T1-w VIBE DIXON | 6.7 | 2.4/ 4.8 | - | 320 x 320 | 380 | 10 | 3 | 470 | axial pelvis and knee |
| ^1^PD-w images were acquired without fat-saturation.  FOV = field of view. PD-w = proton density-weighted. STIR = short-tau inversion recovery. VIBE = volume interpolated breath-hold-examination. | | | | | | | | | |

**Hip MRI in Flexion Abduction External Rotation for assessment of the ischiofemoral interval in patients with hip pain – A feasibility study**

**ELECTRONIC SUPPLEMENTARY MATERIAL**

| **Supplementary Table 2.** Imaging protocol for MRI in neutral and FABER position | | | | | | | | |
| --- | --- | --- | --- | --- | --- | --- | --- | --- |
| Sequence | Repetition  Time  (ms) | Echo  Time  (ms) | Matrix | FOV (mm) | Flip  Angle | Slice  Thickness (mm) | Bandwidth  (Hz/Px) | Image orientation |
| T2 HASTE | 1500 | 91 | 320 x 320 | 350 | 160 | 5 | 710 | axial pelvis |
| T2 True FISP | 1539.4 | 2.47 | 320 x 320 | 250 | 60 | 4 | 521 | axial-oblique hip |
| FOV = field of view. HASTE = **half-fourier-acquired single-shot turbo spin echo sequence.** FISP = fast imaging with steady-state free precession. | | | | | | | | |
